# Assessment of Smear Layer Formation After Caries Removal Using Erbium Laser and Papain-Based Chemo-Mechanical Caries Removal Agent: An In Vitro Scanning Electron Microscopy Study

**DOI:** 10.7759/cureus.47999

**Published:** 2023-10-30

**Authors:** Aditi Dhanvijay, Rajesh Kubde, Pratima Shenoi, Gautam Badole, Shriya Shahu

**Affiliations:** 1 Department of Conservative Dentistry and Endodontics, Vidya Shikshan Prasarak Mandal (VSPM) Dental College and Research Centre, Nagpur, IND

**Keywords:** caries removal, chemo-mechanical agent, erbium laser, smear layer evaluation, minimally invasive dentistry

## Abstract

Introduction

With the advancement in the field of adhesive dentistry, there has been a significant and groundbreaking shift toward the adoption of minimally invasive caries removal techniques where substrate modification is known to enhance adhesive bonding. The smear layer has always been controversial, as its modification helps in bonding, but if contaminated with bacteria, it compromises the formation of a satisfactory marginal seal. Thus, the recognition of the role played by the smear layer highlights the importance of the type of caries removal method employed. Hence, the aim of the present study was to assess and compare smear layer formation after caries removal using an erbium laser and chemo-mechanical caries removal agent by scanning electron microscopy.

Methods

The study involved the evaluation of 30 extracted human molars with occlusal caries. Based on the method of caries excavation, the samples were allocated into two separate groups: group 1 - caries removed using erbium laser (Waterlase, Biolase, Lake Forest, CA); group 2 - caries removed using papain-based chemo-mechanical caries removal agent (Carie Fix, Dengen Dental, Bahadurgarh, India). To assess the smear layer, the samples underwent examination using a scanning electron microscope at 1500x and 3000x magnification after removal of caries. Statistical analysis was done using SPSS version 22 software (IBM Corp., Armonk, NY). Mann-Whitney test was used to compare the mean smear layer (nonparametric) between both groups.

Results

Group 1 (Er:YAG laser) showed significantly greater removal of the smear layer than group 2 (papain-based chemo-mechanical caries removal agent) on the excavated caries surfaces (p < 0.001). In group 1, the dentinal tubules exhibited greater patency when compared to the partial patency observed in group 2.

Conclusion

Both techniques for caries removal were effective; however, the Er:YAG laser method was determined to be more efficient in comparison to the chemo-mechanical agent. These caries removal methods can be considered the future of minimally invasive dentistry.

## Introduction

Dental caries is a major global concern due to its omnipresence and chronicity. The ideology behind its prevention and treatment has evolved significantly in recent decades [1]. The conventional caries removal method undoubtedly offers efficient and faster cavity preparation but does come with several drawbacks. The dentin-pulpal complex is most negatively affected by all rotary cutting techniques, mostly as a result of heat and pressure [2]. An additional drawback of the rotary technique is that it removes both infected and healthy tooth tissue without discrimination [3]. The rotary cutting instruments also leave a thick layer of smear layer behind [4].

As the field of adhesive dentistry has advanced, there has been a significant and groundbreaking shift toward the adoption of minimally invasive caries removal techniques (MICRTs), where substrate modification is known to enhance adhesive bonding [5].

Significant improvements in MICRTs have been made recently with the use of chemo-mechanical agents, laser ablation, sono-abrasion, and air abrasion to remove contaminated tooth tissues. The chemo-mechanical caries removal (CMCR) agents have become popular lately due to their ability to selectively and conservatively remove the carious tooth structure. These agents act by removing the outer visible infected layer of carious dentine while preserving the deeper layer that has the capacity to remineralize. However, these CMCR agents can occasionally be challenging to use in small and inaccessible areas [6]. As a result, when these agents are ineffective, caries are removed mechanically. Conversely, the Er:YAG laser is regarded as a promising approach for caries removal in the realm of operative dentistry. When employed with appropriate parameters, it can precisely ablate the layer of infected enamel or dentin while safeguarding the pulp and surrounding tissues. This method is also reported to be the least traumatic with an effective wavelength (2,940 mm), has zero vibration due to its superficial range, and has a lesser need for administration of local anesthesia. Additionally, it is consistent with minimally invasive preparation that results in clean and micro-retentive surfaces that enhance the bonding of restorations [5]. Since the development of contemporary minimally invasive adhesive dentistry, the morphologic characteristics of the underlying surface as well as the smear layer significantly affect the bonding mechanism. The smear layer is a loosely attached layer formed on the cavity wall after instrumentation consisting of microcrystalline and organic particle debris. This layer can serve as a potential passage for bacterial contamination or microleakage [6]. The modification of the smear layer has been controversial because even though it enhances bonding, its contamination with microorganisms may compromise the formation of a satisfactory marginal seal [7]. Acknowledging the importance of removing this layer, the selection of technique employed for caries removal becomes highly significant. Hence, the current study aimed at assessing the efficacy of erbium laser and papain-based CMCR agents for the elimination of the smear layer through examination with a scanning electron microscope (SEM).

## Materials and methods

An in vitro study was planned following the ethical approval from the Institutional Human Ethical Committee of VSPM Dental College and Research Centre, Nagpur, India (EC/NEW/INST/2020/687).

In the present study, 30 human molars with the extension of carious lesions up to the dentin confirmed using radiographs and with caries involving the occlusal surface were included. Teeth with pulpal pathosis, multi-surface cavities, developmental anomalies, and teeth having cracks, flaws, or any damage from the extraction process were ostracized from the study.

**Figure 1:**
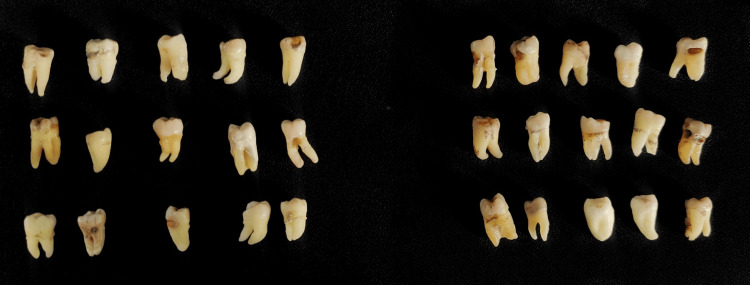
Specimens for group 1 (A) and group 2 (B)

The sample size was calculated using OpenEpi version 3, an open-source calculator. As per the study by Thazhatheethil et al. (2021) [8], considering the mean difference in outcomes in the chemo-mechanical agent and the laser group of 3.85 (2.62) and 0.50 (0.69), respectively, the sample size obtained was 12 teeth with six teeth in each group. As the total sample of 12 was very small, a sample of 30 with 15 teeth in each group was selected as the final sample. The samples were randomly allocated based on the method of cavity excavation into the following two groups (Figure 1): group 1 - caries excavation by using erbium laser (n = 15); group 2 - caries excavation by CMCR agent (n = 15).

Group 1: caries excavation by erbium laser (n = 15)

**Figure 2:**
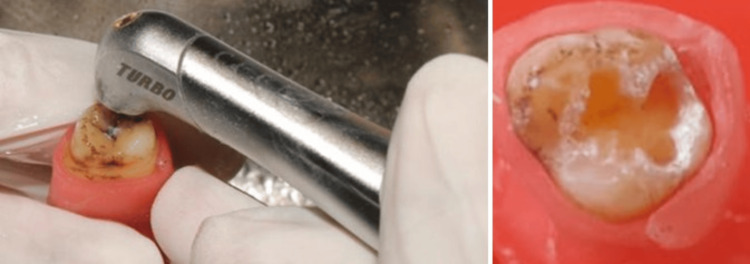
Caries removal using laser

For group 1, caries excavation was performed using an erbium laser (Waterlase, Biolase, Lake Forest, CA) in noncontact mode with water cooling. The process was terminated on encountering hard dentin with a blunt straight probe (Figure 2). The erbium laser used had a wavelength of 2780 nm, pulse energy of 200 mJ, spot size of 1.0 mm, and energy density of 25.5 J/cm2 for 10 pps (pulse per second).

Group 2: caries excavation by chemo-mechanical caries removal agent (n= 15)

**Figure 3:**
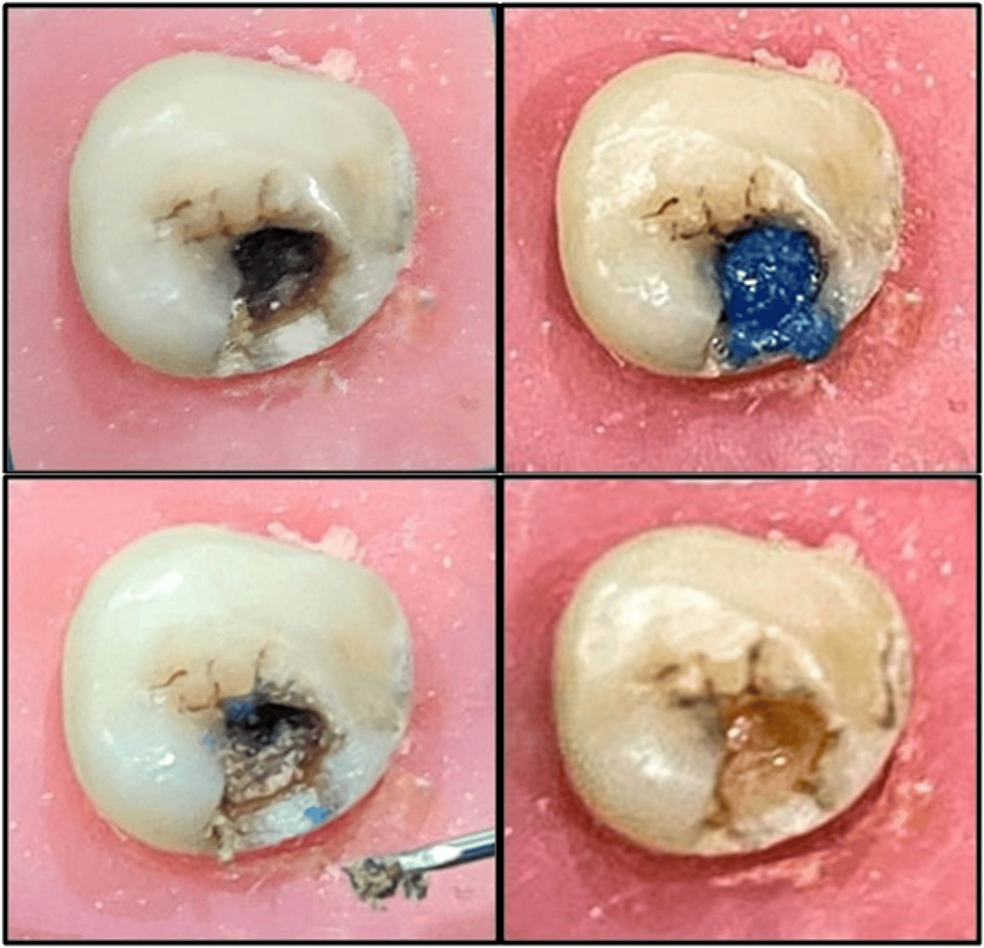
Caries removal using chemo-mechanical caries removal agent

For group 2, caries excavation was performed using Carie Fix (Dengen Dental, Bahadurgarh, India). The agent was administered on the carious surface for a duration of 30 seconds, following the guidelines provided by the manufacturer. The cavity was then washed followed by excavation using a sharp sterile spoon excavator applying gentle pressure. This process was reiterated until the gel no longer contained any debris and a hard cavity surface was encountered (Figure 3).

**Figure 4:**
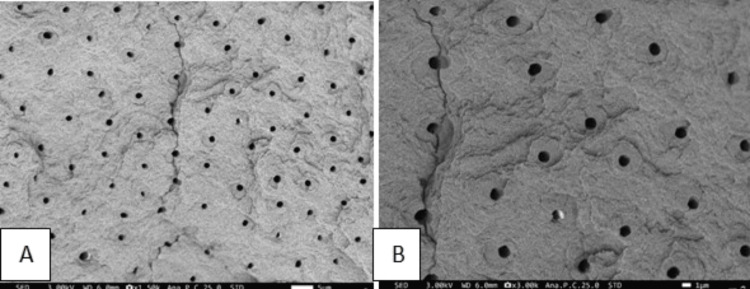
Micrograph representation of group 1 (A) Micrograph at 1500x magnification. (B) Micrograph at 3000x magnification.

**Figure 5:**
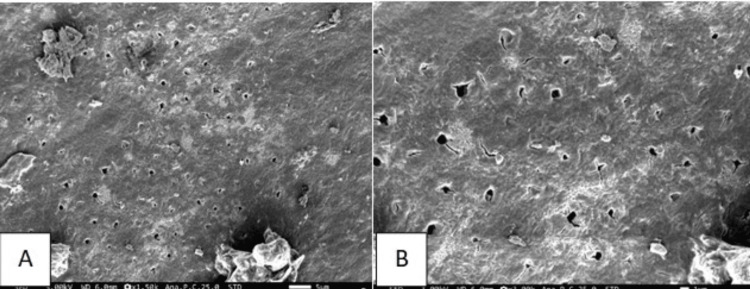
Micrograph representation of group 2 (A) Micrograph at 1500x magnification. (B) Micrograph at 3000x magnification.

The specimens obtained were dried and mounted on brass stubs followed by placement in a vacuum chamber for sputtering. After platinum coating for 40 seconds, they were viewed under SEM for smear layer evaluation (JSM FE-SEM-IT800, White Lab, Chennai, India). At a magnification of 1500x and 3000x, photographic records were made for evaluation of the smear layer (Figures 4, 5).

The assessment of the smear layer was conducted according to the grading system established by Rome et al. [9], as given in Table 1.

**Table 1:** Grading system by Rome et al. Rome et al. [9].

Grade	Interpretation
0	No smear layer with open dentinal tubules
1	Minimal smear layer with >50% visible dentinal tubules
2	Moderate smear layer with <50% visible dentinal tubules
3	Heavy smear layer and obliterated dentinal tubules

Statistical analysis was carried out using SPSS version 22 software (IBM Corp., Armonk, NY). Descriptive statistics, including mean and standard deviation, were computed for quantitative variables, with a predetermined significance level of 0.05 (p < 0.05). The Shapiro-Wilk test was utilized to assess the normality of the data. To compare the percentage of smear layer between the two groups, Fisher's exact test was employed. For the comparison of the mean smear layer, which is a non-parametric measure, between the two groups, the Mann-Whitney test was applied.

## Results

Out of 15 samples in group 1, nine had a score of 0 (no smear layer). The majority of samples in group 2 had a score of 1 (minimum smear layer) and a score of 2 (moderate smear layer). As seen in Table 2, the results obtained were highly statistically significant at 3x magnification (p < 0.001).

**Table 2:** Assessment of the efficacy of erbium laser and chemo-mechanical caries removal agent on smear layer removal at 3000x magnification n = number of samples out of 15 in each group and represented in %. ** Statistically significant (p < 0.001).

Smear layer scoring with description	Group 1 (erbium laser)	Group 2 (chemo-mechanical caries removal)
	n = 15	n = 15
Score 0: No smear layer with open dentinal tubules	9 (60%)	0 (0%)
Score 1: Minimal smear layer with >50% visible dentinal tubules	5 (33.3%)	6 (40%)
Score 2: Moderate smear layer with <50% visible dentinal tubules	1 (6.66%)	7 (46.6%)
Score 3: Heavy smear layer and obliterated dentinal tubules	0 (0%)	2 (13.3%)
Fischer's exact test p-value	p < 0.001**	p < 0.001**

A highly significant statistical difference was found in the two groups at 1.5x magnification using Fischer's exact test (p < 0.001). Out of 15 samples in group 1, eight had a score of 0 (no smear layer). The majority of samples in group 2 had a score of 2 (moderate smear layer) (Table 3).

**Table 3:** Assessment of the efficacy of erbium laser and chemo-mechanical caries removal agent on smear layer removal at 1500x magnification n = number of samples out of 15 in each group and represented in %. ** Statistically significant (p < 0.001).

Smear layer scoring with description	Group 1 (erbium laser)	Group 2 (chemo-mechanical)
	n = 15	n = 15
Score 0: No smear layer with open dentinal tubules	8 (53.3%)	0 (0%)
Score 1: Minimal smear layer with >50% visible dentinal tubules	5 (33.3%)	5 (33%)
Score 2: Moderate smear layer with <50% visible dentinal tubules	2 (13.3 %)	6 (40%)
Score 3: Heavy smear layer and obliterated dentinal tubules	0 (0%)	4 (27%)
Fischer's exact test p-value	p < 0.001**	p < 0.001**

At both magnifications of 1500x and 3000x, group 1 exhibited significantly superior efficacy in removing the smear layer compared to group 2 (p < 0.001).

The outcomes of this in vitro study demonstrated that the erbium laser is more effective at eliminating the infected dentin and the smear layer from carious teeth (p < 0.001) (Table 4).

**Table 4:** Assessment of the efficacy of erbium laser and chemo-mechanical caries removal agent on smear layer removal on mean smear layer removal at 1500x and 3000x magnification. ** p < 0.001 – highly statistically significant difference. Inference: There are highly statistically significant differences present in the smear layer score between group 1 and group 2.

Magnification	Group 1 (laser), mean (SD)	Group 2 (chemo-mechanical), mean (SD)	Mann-Whitney U test	P-value, significance
3000x	0.46 (0.63)	1.73 (0.7)	U = 24.5	p < 0.001**
1500x	0.6 (0.73)	1.93 (0.79)	U =28.5	p < 0.001**

## Discussion

The ability of a dental restoration to last over time largely hinges on the formation of a dependable seal to minimize microleakage and, consequently, the occurrence of secondary caries [10]. The creation of this seal is contingent upon various factors such as the method of tooth preparation and the properties of the adhesive restorative material. The smear layer is also an important factor that influences this seal. Fusayama in 1966 failed to detect bacteria beyond the softened front of dentin in a carious lesion. Therefore, it was established that effective halting of the carious lesion can be achieved by removing the infected dentin. This would preserve the underlying affected dentin without jeopardizing the viability of the pulp and the restoration [11].

This approach ensures the achievement of the optimal peripheral seal using contemporary adhesive dentin bonding agents. The presence of a smear layer can pose challenges to the effectiveness of self-etching primers. This layer has extrinsic proteins and acid-resistant crystals that enter a mineral phase when subjected to a demineralization cycle. The adhesive resin binds to these crystals and proteins if the smear layer is present on the wall. Thus, there is no adhesion of the restoration to the underlying dentin. Additionally, the mineral content of this layer can buffer the acidity of the primer [12].

DH Pashley in 1995 discovered that the resin tag formation was interrupted due to the mineral casts that were present within tubules of affected dentin [13]. These resin tags play a role in enhancing the strength of the resulting bond formed.

The objective of the CMCR technique is to selectively eliminate the outermost infected layer while preserving the affected dentin that has the potential for remineralization and repair. CMCR systems like the novel Carie Fix (papain-based agent) function by degrading the partially deteriorated collagen present in the infected dentin. This minimally invasive technique offers several advantages. It is cost-effective and is generally well-tolerated by patients, thereby minimizing the need for the administration of local anesthesia. Furthermore, it allows for minimal removal of dentin and maximal preservation of healthy tissue during the procedure [9]. Thakur et al. concluded that chemo-mechanical agents utilized for caries removal demonstrated minimal smear layer formation and exhibited patent dentinal tubules [11].

Another minimally invasive technique for caries removal emerged with the advent of lasers. Erbium lasers are the preferred choice for removing carious dentin due to their ability to ablate hard tissues more effectively as a result of their exceptional absorption capabilities. The frequencies of 2.78 and 2.94 um are absorbed primarily by water, which acts as the target chromophore. The water present in the interstitial spaces of the enamel and dentin absorbs the laser energy driving the ablation process. This leads to rapid expansion of this interstitial water and structural disruption in the weakest planes of cleavage [14].

The Er:YAG laser method was used in this study as it is inherently safer, minimally invasive, and minimizes the reliance on local anesthesia. Considering the above factors, Carie Fix and Er:YAG laser were selected for caries removal and smear layer assessment in the present study. The results of the present study depicted that carious dentin ablated by the Er:YAG laser displayed a rugged, irregular, and scaly surface devoid of the smear layer. The dentinal orifices did not exhibit any widening but remained patent. Moreover, the peritubular dentin showed slightly more protrusion than the adjacent intertubular dentin. The cavity floor having surface irregularities, devoid of a smear layer, provides an optimal surface for the formation of a strong bond with adhesive restorative materials. It may additionally benefit by providing an etching behavior not observed with mechanical or chemo-mechanical systems.

In addition to the positive outcomes observed with laser ablation, surface microcracks were also noted. Katsumi et al. documented that the use of an erbium laser causes substructural cracks in dentin [15]. Other researchers have also highlighted the formation of cohesive fractures more commonly in dentin with the use of laser [16-21]. Structural weakening caused by the laser is not just restricted to the superficial layer of dentin, but it also affects and weakens dentin up to a depth of 3-5 mm. This further enhances the adhesion of adhesive materials. Consequently, the potential compromise in dentin integrity needs to be considered when evaluating the clinical application of laser ablation techniques.

The surface treated with Carie Fix showed several undermined areas with uneven surfaces and moderate smear layer formation. Dentinal tubules showed partial patency and were observed to be covered with residues of the smear layer. Carie Fix treatment modified the dentinal surface to a more granular and rough topographic structure comparable to dentin prepped with an erbium laser. These findings were not in accordance with the results of the study by Avinash et al., wherein they obtained a minimal smear layer after using a CMCR agent [22]. The disparity in the research findings may arise from the use of different agents utilized in the studies.

The sodium hypochlorite-based agent CariSolv was used in their study compared to the present study that used a papain-based agent. Sodium hypochlorite causes chlorination by producing chloramines after reacting with amino acids. Consequently, hydroxyproline undergoes a conversion into pyrrole-2-carboxylic acid, initiating the breakdown of partially degraded collagen fibers, which, in turn, results in the softening of the outer layer of carious dentin [23].

On the other hand, the Carie Fix agent was used in this study, which is based on papain, an enzyme similar to human pepsin. Papain has several benefits due to its anti-inflammatory and debriding properties. It enhances the healing process, has bacteriostatic and bactericidal effects, and preserves the underlying healthy tissues as it acts specifically on carious tissue, which is devoid of alpha-1 antitrypsin. As healthy tissue contains alpha-1 antitrypsin, it is not affected by papain [16].

In recent times, the traditional “extension for prevention” approach has been largely replaced by "prevention of extension” in modern dentistry [24]. This shift emphasizes the preservation of maximum tooth structure and aims to minimize the extent of destruction. As per the findings of this study, Er:YAG laser and Carie Fix present several advantages within the framework of minimally invasive dentistry concepts. In recent times, there has been an expansion in the range of cavity preparation methods.

Numerous novel techniques have emerged that require in-depth evaluation, as they have the potential to significantly impact the clinical outcome of adhesive techniques. Laser-assisted caries excavation using Er:YAG lasers and the chemomechanical approach for caries removal show promise as the future of noninvasive techniques in dentistry. However, further in vivo examination and research are necessary to thoroughly investigate these new methods and understand their effects on adhesive procedures before introducing them into regular clinical practice.

When the limitations of the present study are considered, not all surface characteristics were addressed. Further, because of the small sample size, it is difficult to make a definite conclusion. Factors related to patients were also not reported since it is an in vitro study and hence, additional research, encompassing in vivo experiments, is imperative to authenticate the discovered results. Hence, future studies with patient-related assessment factors are advised to be carried out.

## Conclusions

Both laser irradiation and the use of chemo-mechanical agents are effective for caries removal with minimal adverse effects. Therefore, they can be considered as a successful alternative to conventional methods of caries removal. Nevertheless, the Er:YAG laser was discovered to be more effective than the chemo-mechanical agent based on papain for caries excavation. It was a consistent finding that with minimal invasive preparation, minimal smear layer and exposed dentinal tubules aid in strong micro-retention, ideal for adhesive restorations. An in vivo research comparing the two techniques of removal of dental caries should be carried out to assess the patient factors that this study could not address.
